# Transcatheter Aortic Valve Implantation to Treat Degenerated Aortic, Mitral and Tricuspid Bioprosthesis

**DOI:** 10.3390/jcm13020592

**Published:** 2024-01-19

**Authors:** Arif A. Khokhar, Jonathan Curio, Alessandro Sticchi, Adam Hartley, Ozan M. Demir, Neil Ruparelia

**Affiliations:** 1 Cardiology, Hammersmith Hospital, Imperial College Healthcare NHS Trust, London W12 0HS, UK; arifkhokhar@doctors.org.uk (A.A.K.); adam.hartley@nhs.net (A.H.); 2 Department of Cardiology, Heart Center Cologne, Faculty of Medicine, University Hospital, University of Cologne, 50937 Cologne, Germany; jonathan.curio@uk-koeln.de; 3 Cardiac Catheterisation Laboratory, Cardiothoracic and Vascular Department, Azienda Ospedaliero Universitaria Pisana, 56126 Pisa, Italy; sticchialessandro@gmail.com; 4 Università di Pisa, Lungarno Pacinotti 43, 56126 Pisa, Italy; 5 Department of Cardiology, Essex Cardiothoracic Centre, Mid and South Essex NHS Foundation Trust, Basildon SS16 5NL, UK; 6 Cardiology, Royal Berkshire Hospital, Reading RG1 5AN, UK

**Keywords:** TAVI, bioprosthetic failure, VIV, valve-in-valve

## Abstract

Transcatheter aortic valve implantation (TAVI) is now well established as the treatment of choice for patients with native aortic valve stenosis who are high or intermediate risk for surgical aortic valve replacement. Recent data has also supported the use of TAVI in patients at low surgical risk and also in anatomical subsets that were previously felt to be contra-indicated including bicuspid aortic valves and aortic regurgitation. With advancements and refinements in procedural techniques, the application of this technology has now been further expanded to include the management of degenerated bioprosthesis. After the demonstration of feasibility and safety in the management of degenerated aortic bioprosthetic valves, mitral and tricuspid bioprosthetic valve treatment is now also well-established and provides an attractive alternative to performing redo surgery. In this review, we appraise the latest clinical evidence and highlight procedural considerations when utilising TAVI technology in the management of degenerated aortic, mitral or tricuspid prosthesis.

## 1. Introduction

Transcatheter aortic valve implantation (TAVI) is now well established as the treatment of choice for patients with native aortic valve stenosis who are high- or intermediate-risk for surgical aortic valve replacement [1]. Recent data has also supported the use of TAVI in patients at low surgical risk [2,3] and also in anatomical subsets that were previously felt to be contra-indicated including bicuspid aortic valves [4] and aortic regurgitation [5].

With advancements and refinements in procedural techniques, the application of this technology has now been further expanded to include the management of degenerated bioprosthesis. After the demonstration of feasibility and safety in the management of degenerated aortic bioprosthetic valves, mitral and tricuspid bioprosthetic valve treatment is now also well established and provides an attractive alternative to performing redo surgery.

In this review, we appraise the latest clinical evidence and highlight procedural considerations when utilising TAVI technology in the management of degenerated aortic, mitral or tricuspid prosthesis.

## 2. TAVI for Degenerated Surgical Aortic Bioprosthetic Valves: Valve-in-Valve (ViV)-TAVI

Transcatheter aortic valve-in-valve implantation (ViV-TAVI) is safe and effective for treating degenerated surgical bioprosthetic aortic valves (SAV) [6,7,8,9]. Historically, treatment options for SAV degeneration were limited to high-risk redo surgery or conservative management due to the advanced age and associated co-morbidities for this cohort of patients. However, in recent times, following demonstration of the safety and efficacy of the VIV-TAVI approach, this is increasingly being adopted as the first-line treatment in this patient group. The widespread adoption of this treatment modality has been observed in the STS-ACC TVT registry, where ViV-TAVI represented 6.14% of all TAVI procedures being performed as of 2019 [10]. The growth in ViV-TAVI procedures has mirrored, and to an extent promoted, the increase in the utilization of bioprosthetic over mechanical surgical valves at the index surgical intervention, as patients now have a viable alternative lifetime management strategy for aortic stenosis [11]. Accordingly, ACC/AHA guidelines have adjusted their guidance proposing mechanical prosthesis to be considered in patients age <50 years and a biological prosthesis can be considered between ages 50 to 65 years as part of a shared decision making process [12].

Initial data on ViV-TAVI outcomes have been described in the prospective multinational VIVID registry, which included 459 patients, receiving either a balloon-expandable (BEV, Sapien, Edwards LifeSciences, USA) or self-expandable (SEV, predominately Evolut, Medtronic, USA) TAVI [6]. The 30-day and 1-year mortality rates were 1.7% and 16.8%, with a higher mortality rate observed in patients with smaller SAV prostheses and in those where stenosis was the relevant mode of failure. The stroke rate at 30 days was 1.7%. These clinical results have subsequently been confirmed by other large registries, using either BEV or SEV, as well as in cohorts with a mixed usage of device approaches [7,8,9] (Table 1). In the largest propensity-matched analysis comparing ViV-TAVI against redo AVR, ViV-TAVI was associated with lower 30-day mortality (7.5% absolute risk reduction), improved five-year survival (76.8% versus 66.8%) and lower rates of post-operative complications including the need for blood transfusions, pacemaker implantations and length of hospital stay.

### 2.1. Pre-Procedural Considerations for ViV-TAVI

#### 2.1.1. Type of Surgical Aortic Bioprosthesis

**Figure 1:**
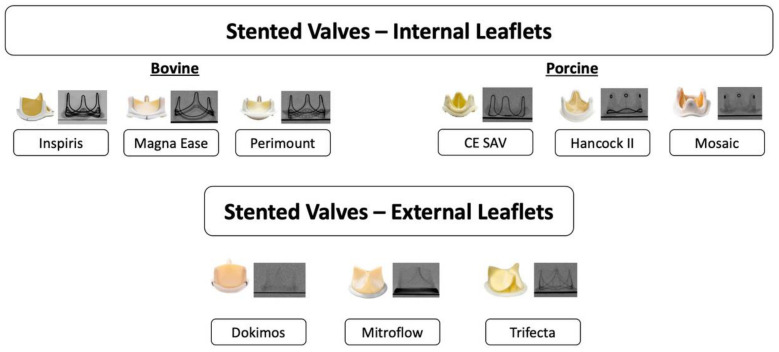
Types of surgical bioprosthetic aortic valves. Surgical bioprosthetic aortic valves (SAV) vary according to the tissue composition of their leaflets and whether the leaflets are mounted externally or internally to the stent frame, which impacts the fluoroscopic visualization of the valve.

There is a broad spectrum of surgical bioprosthetic valves available and knowledge about their different features is key when assessing a patient with a failed surgical valve and planning for a ViV-TAVI procedure (Figure 1). In general, SAVs may be stented or stentless, with the latter representing around one fifth of failing surgical valves, and do not feature any fluoroscopic markers [6]. Stented SAVs can be further subdivided into those with internally mounted or externally mounted leaflets and a further category of sutureless self-expandable prostheses. Internally mounted leaflets may result in a shorter leaflet skirt being created when the SAV leaflets are pinned up and outwards during ViV-TAVI, whilst externally mounted leaflets may reach higher up and extend outwards, increasing the risk of coronary obstruction or impaired coronary access [14]. Furthermore, with internally mounted porcine leaflets the resulting true internal diameter (ID) after displacement of the leaflets by TAVI may be smaller (true ID around 2 mm less than nominal) than when bovine pericardial leaflets (true ID around 1 mm less than nominal) are displaced, whilst with externally mounted leaflets the true internal diameter equals the nominal stent value. If no previous information is available, identification of the implanted SAV may be possible based on the radiopaque appearance of the valve seen on pre-procedural CT.

#### 2.1.2. Pre-Procedural Assessment

**Figure 2:**
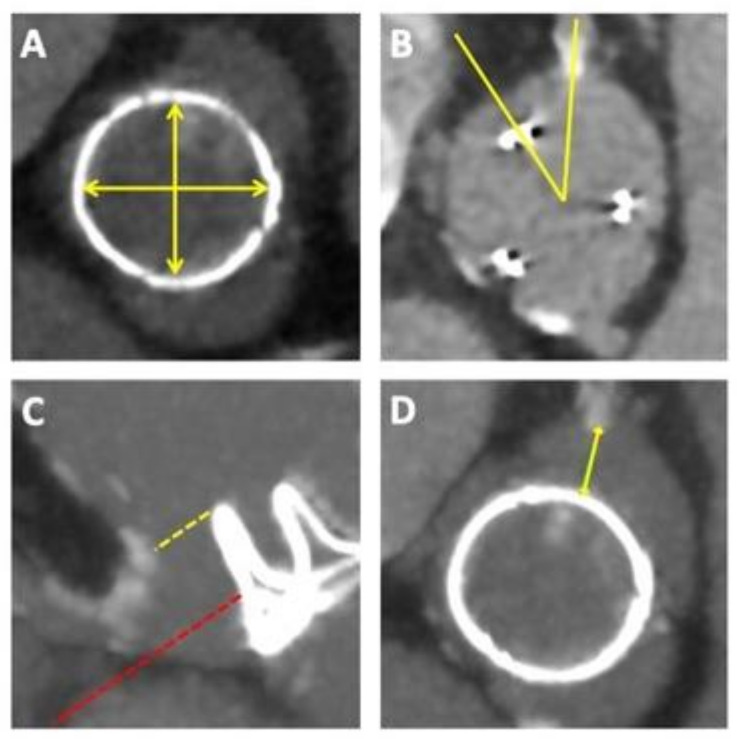
Pre-procedural planning for ViV-TAVI. Pre-procedural computed tomography is required to evaluate the (**A**) true internal dimensions of the implanted SAV, (**B**) the alignment of the SAV with respects to the native coronaries, (**C**) the coronary and sinus heights in relation to the top of the SAV frame, or if no posts are visible, then anticipated heights of leaflets and (**D**) the gap between the frame and/or leaflets and coronary ostia to estimate the risk for coronary obstruction.

In addition to understanding the type and size of the implanted bioprosthesis, several other key parameters obtained from the pre-procedural CT are of relevance (Figure 2). Although sizing is generally based on the size and type of the surgical bioprosthesis being treated, precise measurements of the implanted bioprosthesis should be performed to confirm the true ID. In general, it is important to understand and evaluate the relationship between the ViV-TAVI complex comprising the surgical and the transcatheter valves as well as the neoskirt created by the displaced SAV leaflets and the surrounding anatomy, due to the risk of acute or delayed coronary obstruction, which although rare (3–5%) can be fatal [14,15]. The risk of coronary obstruction is greater when treating stentless or stented valves with externally mounted leaflets, using SEV TAVI platforms [15]. The two principal mechanisms underlying coronary obstruction, include direct obstruction of the coronary ostia by the pinned surgical leaflet or indirect obstruction to coronary flow due to sinus sequestration caused by the pinned-up surgical leaflet rising above the sinotubular junction (STJ). In general, the risk of coronary obstruction is greater in narrow and shallow aortic roots where the ViV-TAVI complex lies in close proximity to the coronary ostia. For coronary ostia located above the SAV leaflets the risk of obstruction may be negligible. For coronary ostia arising below the level of the SAV posts or pinned-up leaflets, the distance between the implanted valve and coronary ostia, valve-to-coronary (VTC) becomes relevant. A retrospective evaluation of 37 coronary obstruction cases from the VIVID registry, identified and validated a cut-off VTC measurement of 4 mm to identify those at high-risk for coronary obstruction [14]. A further relevant parameter may be the valve-to-STJ distance (at, as well as, 2 mm above and below the sinotubular junction), with <2.5 mm being considered high risk and a distance between 2.5 mm and 3 mm being considered borderline risk for coronary obstruction [16].

In addition to coronary obstruction, which is rare but potentially fatal, impaired coronary access is increasingly relevant as ViV-TAVI emerges as a potential lifetime management strategy particularly for younger patients with aortic stenosis [17,18]. Challenging coronary access is anticipated following ViV-TAVI particularly in narrow and shallow aortic roots. Unlike, coronary obstruction, specific validated measurements predicting the feasibility of coronary access do not exist but theoretically a VTC and VTSTJ < 2 mm is said to result in very challenging future coronary access. However, it should be noted that these theoretical values do not fully capture the three-dimensional sinus space, which may facilitate the use of dedicated valve-specific cannulation techniques [19,20,21].

### 2.2. Procedural Considerations

A key consideration during ViV-TAVI is the optimization of the haemodynamic result. Following SAVR, patient–prosthesis mismatch, which is a risk factor for early valve degeneration, is prevalent in up to 45% of patients [22,23]. Furthermore, following ViV-TAVI, the presence of high residual gradients has a significant impact on 1-year mortality rates, particularly in patients with smaller SAV and pre-existing PPM [6,7,24]. Therefore, the goal during ViV-TAVI is to ensure an optimal haemodynamic result, with low residual gradients; while acknowledging that the pursuit of this goal may have to be balanced against the safety of doing so. The first step is the choice of TAVI prosthesis, with supra-annular SEV consistently demonstrating superior haemodynamic outcomes following ViV-TAVI, particularly in the setting of a small SAV. The recent LYTEN trial randomized 102 patients with smaller failed SAVR (i.e., ≤23 mm), thus, likely prone to higher post-procedural gradients and patient–prosthesis mismatch, to either a balloon-expandable (Sapien) or a self-expandable (Evolut) platform [13]. There were no clinical differences at 30 days; however, based on echocardiographic assessment, SEV was associated with significantly lower mean and maximum transvalvular gradients (15 vs. 23 mmHg and 28 vs. 40 mmHg) as well as a tendency towards less PPM (44% vs. 64%) when compared to a BEV.

Following ViV-TAVI, if residual gradients are elevated then balloon post-dilatation is recommended. If gradients are still elevated then balloon valve fracture (BVF) can be considered, whereby a high-pressure balloon inflation is performed to fracture the sewing ring of the SAV. BVF can be performed before or after ViV-TAVI. BVF before TAVI may ease implantation of SEV, while it may cause significant acute aortic regurgitation and post-dilatation might still be needed. BVF after TAVI may further improve expansion of the transcatheter prosthesis, while it can, on the other hand, risk migration of the TAVI device and potential harm to its leaflets [25,26]. It should be noted that not all SAVs can be fractured, and this should be checked beforehand. Furthermore, different balloons and valves have different recommended inflation pressures and rated burst pressures (RBP) to achieve BVF.

During ViV-TAVI, the implant depth is determined fluoroscopically using the radiopaque suture line of the SAV marking the landing zone, with the ViV application providing guidance on the optimal landing zone for each combination of ViV-TAVI. Furthermore, several SAVs feature radiopaque markers of the mounted commissures. With tall-frame SEVs, commissural alignment is important both in terms of preserving future coronary access and in ensuring leaflet modification (see below) procedures are effective at reducing the risk of coronary obstruction. Data on SAVs show that surgeons try to achieve an optimized commissural alignment, even correcting for coronary eccentricity [27]. Thus, the orientation of the SAV should be the target for ViV-TAVI alignment and, similar to the cusp-overlap technique in native TAVI targeting one isolated commissure, for ViV one of the commissural SAV posts should be isolated and used as a reference for rotational TAVI orientation and deployment [28].

In cases of the increased risk for coronary obstruction, adjunctive techniques, such as BASILICA (bioprosthetic or native aortic scallop intentional laceration to prevent iatrogenic coronary artery obstruction) or chimney-stenting (implantation of a long coronary stent protruding from the coronary ostium to the ascending aorta above the risk level) may be performed [29,30,31].

### 2.3. Future Considerations Regarding ViV

The number of ViV-TAVI procedures is set to further increase in the future as SAVR and subsequent TAVI represents one relevant pathway of the lifetime management in patients with aortic stenosis initially treated with a bioprosthetic valve [17,18].

## 3. TAVI for Degenerated Transcatheter Aortic Valves: Redo TAVI

The long-term durability of transcatheter aortic valves (TAV) remains unknown, but rates of bioprosthetic valve failure (BVF) for TAV at 8–10 years follow-up remains low and equivalent to surgically implanted bioprosthetic aortic valves [32]. Globally, the median age of patients undergoing TAVI is decreasing, a trend which is expected to continue over the coming decades [33,34]. Therefore, as TAVI expands to younger patients who are likely to outlive the durability of the index implanted TAV, it is expected that a growing number of failed TAVs will be encountered. If the cause for TAV degeneration is isolated structural valve deterioration (SVD), redo TAVI is likely to be the treatment option of choice [35].

### 3.1. Clinical Experience

Redo TAVI represents around 0.3–0.5% of total TAVI procedures being performed currently. Although limited data and long-term follow-up are available, initial experience demonstrates acceptable rates of procedural safety, device success and clinical outcomes [36,37,38,39,40] (Table 2).

In the largest published series to date of 1320 redo TAVI procedures being performed using a balloon-expandable valve (BEV), procedural complication rates were low, whilst rates of stroke and mortality at both 30 days and 1-year were similar between native TAVI and redo TAVI populations [42]. In the redo TAVR registry, 212 consecutive redo TAVI procedures were equally performed with the use of either a self-expandable valve (SEV) or balloon-expandable valve (BEV). A BEV-in-SEV approach was used in 56/212 (26%) procedures, whilst a SEV-in-BEV approach was adopted in 31/212 (15%) procedures [41]. The overall 30-day mortality and procedural safety was similar irrespective of whether the first or second THV was a BEV or a SEV, but procedural success was higher if the second THV implanted was a SEV (77.2%) compared to a BEV (64.3%) [43]. This was driven by the lower residual gradients when the second THV used was a SEV (10.3 mmHg (8.9–11.7 mmHg) vs. 15.2 mmHg (13.2–17.1 mmHg)).

An alternate treatment option to redo TAVI is surgical explantation of the TAVI prosthesis and surgical aortic valve replacement. A comparison of 257 redo TAVI procedures with 130 surgical TAVR explants performed over a similar time period from the US Medicare database demonstrated a significantly lower 30-day mortality (6.2% vs. 12.3%) and lower incidence of in-hospital major bleeding, acute kidney injury, and a shorter duration hospital stay (median 6 vs. 10 days) with redo TAVI.

### 3.2. Principles of Redo TAVI

One of the key challenges associated with redo TAVI, is the numerous different combinations and configurations of redo TAV possible. Each THV can be implanted within either itself or inside a different THV, in a different sequence, with different implantation depths and degrees of alignment results. For simplification, redo TAVI combinations can be categorised into the following four groups based on TAV design: short-in-short, short-in-tall, tall-in-short, tall-in-tall. An understanding of how the two sets of THV frames and leaflets interact is required to better understand the principles underpinning redo TAV.

#### 3.2.1. Leaflet Neoskirt

**Figure 3:**
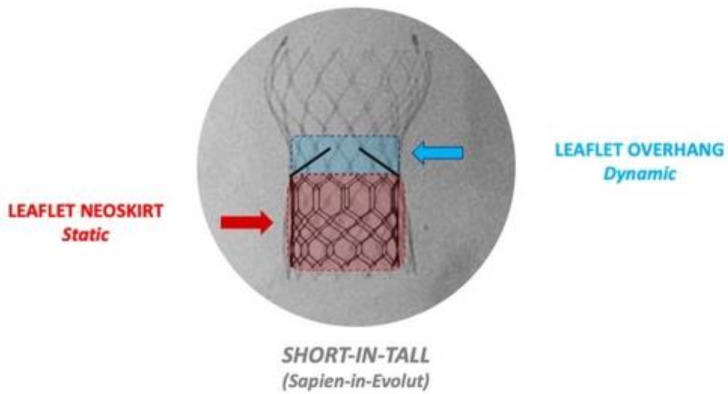
Terminology for redo-TAVI, An example of a short-frame balloon-expandable valve implanted in a low position inside a tall-frame supra-annular self-expandable valve. This creates (1) leaflet neoskirt, which refers to the pinned up leaflets of the first valve, and (2) leaflet overhang, which refers to the residual overhanging supra-annular leaflets.

During redo TAVI, the leaflets of the first degenerated THV are pinned upright by the frame of the second THV. As the leaflets of the first THV are pinned back, a cylindrical barrier, in a fashion similar to a covered stent, is be created. The vertical distance from the inflow of the first THV to the top of these pinned back leaflets is referred to as the *neo-skirt* (Figure 3). Tall-framed supra-annular self-expandable valves (SA-SEVs) have their higher positioned leaflets and therefore would be expected to have a higher or taller neo-skirt in comparison to a short-framed intra-annular balloon-expandable valve (BEV) [44,45]. If the leaflet neoskirt arises above the plane of the coronary ostia or STJ, then there is an increased risk of direct coronary obstruction or indirect coronary obstruction due to sinus sequestration, if the VTC or VTSTJ dimensions are narrow.

#### 3.2.2. Leaflet Overhang

If redo TAVI is performed with the combination of a short-frame THV, e.g., Sapien XT/3 inside of a tall-frame THV with supra-annular positioned leaflets, e.g., Evolut/Corevalve, and the outflow portion of the short frame lies below the supra-annular leaflets, this would result in residual leaflet tissue overhanging the second THV, described as leaflet overhang [45] (Figure 3). At present, the impact of leaflet overhang on subsequent haemodynamic outcomes, leaflet thrombosis and long-term durability remains unknown, with early bench data suggesting that leaflet overhang has no consequence on subsequent hydrodynamic function in terms of mean gradient or effective orifice area [46,47].

#### 3.2.3. Frame Anchoring

Appropriate anchoring and sealing of the second THV is critical to ensuring optimal haemodynamics and avoid migration or embolization. Although in vitro recommendations exist for treatment of degenerated BEV and SEV [48,49], in vivo sizing may be different due to THV frame under-expansion, which can be evaluated on pre redo-TAVI CT [50]. A retrospective evaluation of 328 post-Sapien S3 implantation CT scans, demonstrated significant under-expansion of the implanted Sapien S3 in 92.1% of patients. Using an under-expansion threshold of <89%, the sizing strategy for redo TAVI would be different in up to 13% of patients.

#### 3.2.4. Frame Alignment

When redo TAVI is being performed using a SEV as either the first and/or second THV, then care should be taken to ensure commissural alignment of the SEV [51]. This is important to facilitate future coronary re-access, a key consideration in younger patients with longer life-expectancies and can facilitate future need for leaflet modification. The long-term consequences on the haemodynamics and valve durability with different degrees of commissural alignment between two THVs is unknown. Similar to commissural alignment, cell alignment can be important particularly with tall-frame THVs, to avoid creating a metallic mesh or net of cells impermeable to a coronary catheter thereby preventing future coronary re-access [44].

#### 3.2.5. Frame Distortion

The ability to modify or distort the frame of the first THV during placement of the second THV is an important consideration and depends on the design and metallic properties of the different valve frames. Bench data has shown that placement of a BEV inside of a SEV can lead to outward displacement of the SEV valve frame [46,47]. Furthermore, this degree of outward displacement varies depending on the design and size of the first THV and the design, size and implantation depth of the second THV [46].

The properties of the leaflet neoskirt, leaflet overhang and extent of frame anchoring, alignment and expansion are all dictated by the type, sequence, implantation depth and alignment of the two THVs implanted [45,46,47,52]. The resulting geometrical interaction between the assembled valvar complex with the aortic annulus, aortic sinuses and coronary ostia plays an essential role in the safety and efficacy of redo TAVI.

### 3.3. Pre-Procedural Planning for Redo TAVI

**Figure 4:**
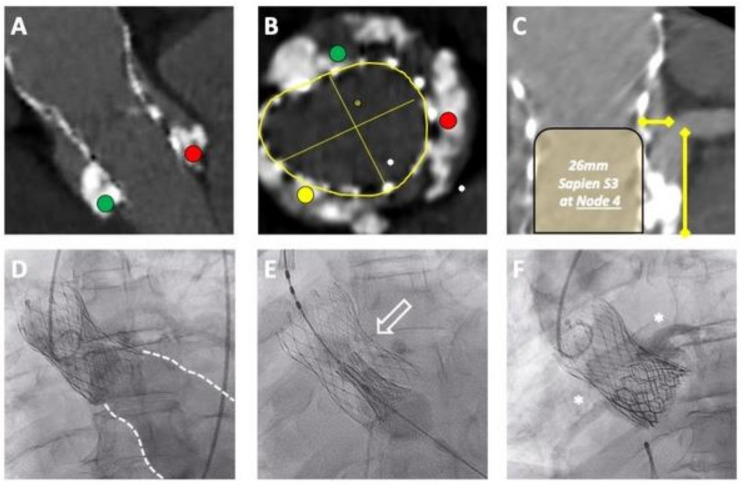
Planning and performing a redo-TAVI procedure. An example of a redo-TAVI procedure where a balloon-expandable Sapien S3 Ultra (Edwards Lifesciences, USA) TAV was used to treat a degenerated self-expandable Evolut R 34 mm TAV. (**A**) Presence of heavy calcification located at the right cusp (green dot) and left cusp (red dot) contributed to underexpansion of the first TAV, which impacts the (**B**) sizing strategy of the S3. (**C**) A low implantation targeting node 4, was deemed safe to prevent coronary obstruction. (**D**–**F**) Fluoroscopic images demonstrating the (**D**) extent of regurgitation pre-procedurally, (**E**) targeting node 4 (white arrow) and (**F**) final result confirming patency of both coronary ostia (represented by stars).

Similar to native-valve TAVI, pre-procedural computed tomography (CT) plays a key role in guiding the selection of THV type, size and implantation strategy [48,50]. Particular attention must be paid to the intended combination and configuration of the valvar complex and its relationship with the surrounding aortic root anatomy to avoid the risk of coronary obstruction or unfeasible coronary access (Figure 4).

#### Index THV

The first step is to evaluate the baseline aortic root, annular and LVOT dimensions, coronary heights and establish the presence and severity of any annular or LVOT calcification on the pre-index TAVI CT scan. Oversizing the second THV with respect to the native annular dimensions, particularly with a BEV, may theoretically increase the risk of aortic root injury or rupture. Similarly, in the presence of severe calcification, aggressive overexpansion of the first THV by a BEV may risk injuring the aortic root and LVOT complex.

The pre-redo TAVI CT can then be used to evaluate the geometry of the first THV. The implant depth, internal diameter, valve expansion and commissural alignment, if possible, should be evaluated. The geometrical relationship between the first THV with the surrounding aortic anatomy and coronary heights then needs to be evaluated. The key structures are the coronary ostia, SoV and STJ and the gap between these aortic structures and the frame of the first THV. For tall-frame THVs, these measurements should be performed at different levels, where the second THV may be implanted.

Based on the analysis of the first THV, its dimensions and degree of expansion, the second THV should be selected accordingly. Sizing algorithms have been proposed depending on the combination and sequence of valves being implanted [48,49]. The current consensus is to generally match or slightly over-size the second THV; however, under-expansion of the index THV may alter the sizing strategy [50].

The final step involves planning the implantation depth of the second THV. The higher the implantation depth, the taller the neoskirt [45]. Therefore, the relationship between the neoskirt and the coronary arteries and STJ should be simulated at different implantation depths to predict the risk of coronary obstruction [53]. Consideration can also be given to the expected leaflet overhang that may occur at different implantation depths. The impact these stenotic stiff leaflets may have on in vivo haemodynamics, leaflet thrombosis and ultimately valve durability remains unknown. In this scenario, the options would be to either accept the leaflet overhang, or to attempt leaflet modification of the first THV to facilitate a higher implantation of the second THV.

Given the complex and multi-parametric nature of planning required for redo TAVI, computational modelling programmes have been developed that allow for patient-specific procedural planning [54]. Although awaiting comprehensive validation, these software programmes can help in the 3D visualisation of the geometrical interaction between different redo TAVI combinations and configurations with the surrounding anatomy.

### 3.4. Leaflet Modification for Redo TAVI

In challenging anatomical scenarios such as low coronary heights and narrow short STJ dimensions, pre-procedural planning may indicate a high risk of sinus sequestration or coronary obstruction following redo TAVI. In these select cases, leaflet modification may be considered as an adjunctive procedure to reduce the risk of coronary obstruction if surgical intervention is deemed to be associated with prohibitive risk.

Leaflet modification with BASILICA has been shown to be an effective strategy to reduce the risk of coronary obstruction in the setting of native-valve TAVI or ViV-TAVI. However, to date, only isolated case reports exist describing the safety and efficacy of BASILICA to prevent coronary obstruction following redo TAVI [55,56,57,58]. Additionally, the effectiveness of leaflet splitting might be limited if the commissures of the first TAV are misaligned with respect to the native coronary ostia.

### 3.5. Coronary Access after Redo TAVI

At present there are no clinical studies to evaluate the feasibility or challenge of coronary access following redo TAVI. Previous CT-based simulation studies have hypothesised that coronary access after redo TAVI may be unfeasible in up to 50% of cases, particularly with the use of SA-SEVs [59,60]. These studies have suggested that coronary access may be unfeasible when coronary arteries arise below the level of the neoskirt and the gap between the valve frame and aorta or STJ is <2 mm (the size of a 6Fr catheter).

Given the importance of the neoskirt to coronary access, it follows therefore that altering the neoskirt geometry by changing the THV type, size sequence and/or implantation depth can all have an impact on coronary access [44,50,61]. For example, coronary access would be expected to be easier following BEV-in-BEV with a Sapien valve as compared to SEV-in-SEV with an Evolut valve, due to the shorter valve frame and lower position of the leaflets and subsequent neoskirt. When implanting a BEV inside of a SEV, altering the implantation depth of the BEV may have a significant impact on the neoskirt and subsequent coronary access.

Furthermore, the degree of alignment between the two THVs can also have a significant impact. If the two commissural posts of a tall-frame SEV are misaligned, then there is a greater surface barrier which must be circumnavigated by a catheter. Similarly, if the cells of a tall-frame SEV, such as an Evolut, are not properly aligned, then a dense mesh of cells would be created which could further increase the challenge of coronary access [44].

### 3.6. Future Perspectives

As TAVI continues to expand towards younger patients and redo TAVI procedures become more frequent, there will be a need to develop dedicated valve-in-valve platforms to facilitate reliable, reproducible and durable redo TAVI procedures to be performed. Device technology and implantation techniques should be refined to ensure that optimal valve expansion and commissural alignment can be reliably and consistently achieved. A patient-specific approach should be adopted to determine the optimal implantation depth during index TAVI, that would facilitate a redo TAVI procedure, particularly in younger patients.

## 4. TAVI for Treatment of Degenerated Mitral Bioprosthesis

The most common valvular disease in the Western world is mitral valve disease, for which surgery is the gold standard treatment [62,63]. Surgical mitral valve repair is preferred to mitral valve replacement, owing to favourable comparative clinical outcomes and postponing the need for mitral valve replacement, which can be performed using either a bioprosthetic or a mechanical valve [64]. The use of a mechanical valve is preferable in younger patients due to its lifelong durability; however, with the drawback of requiring lifelong anticoagulation. In contrast, for elderly patients, a bioprosthetic (pericardial or porcine) valve is the preferred option as long-term anticoagulation is not required. However, the durability of bioprosthetic valves varies from 10 to 15 years in the mitral position due to their progressive degradation over time [65]. In such patients, transcatheter mitral valve replacement (TMVR) is an emerging alternative therapy to redo surgical intervention. TMVR can either be performed using dedicated valve platforms or using existing TAVI devices (e.g., Sapien 3) with a modified implantation technique. In this section we outline the current evidence for TMVR in patients with failed surgical mitral valve repair (mitral ring) or degeneration of bioprosthetic valves and highlight practical procedural considerations for performing valve-in-valve (ViV) or valve-in-ring (ViR) procedures using existing TAVI platforms.

### 4.1. Current Evidence

The largest study of mitral ViV and ViR to date demonstrated that TMVR provided excellent outcomes for patients with degenerated bioprosthetic valves but TMVR for failed annuloplasty rings (ViRs) was associated with a greater risk of procedural complications [66,67,68]. In the seminal study by Yoon SH et al., 322 patients underwent TMVR for degenerated mitral bioprosthetic valves (ViVs) and 141 patients for failed annuloplasty rings [66]. The majority of patients were deemed to be at high risk for conventional surgery with a mean Society of Thoracic Surgeons (STS) score of 9.0%, without significant differences across the groups (ViV vs. ViR: 9.2  ±  7.2% vs. 8.1  ±  6.4%). For mitral ViVs, the procedural success rate was 74% and a technical success rate of 94.4%. In view of the fact that rates of in-hospital death for a second mitral replacement procedure range from 9% to 12.6%, and that a significant percentage of the initial mitral regurgitation (MR) in the study had a functional aetiology, the thirty-day mortality rate of 6.2% is acceptable [69,70,71]. In addition, previous studies on ViV TMVR reported a comparable 30-day mortality of 6.7–11.4% [67,68]. Major left ventricular outflow tract (LVOT) obstruction occurred in 2.2% of patients, less than 1% required emergent surgery, and 3.3% had severe paravalvular leakage (PVL) at 30-day echo; in total the frequency of post-procedural adverse events was very low [72]. For Mitral ViR, procedural success rates were 57.4%, 5% LVOT obstruction, and significant PVL of 18.4% immediately after the procedure and 12.6% at 30 days following the procedure (the primary predictor of death at follow-up). Mortality at thirty days was 9.9% and 30.6% at 1 year. This was probably due to lower left ventricular function in the ViR group. This is comparable to 1-year mortality rates after mitral valve reoperation of 23.1%, mainly related to high in-hospital mortality (12.6%) [70].

### 4.2. Procedural Considerations

For TMVR the main procedural considerations are patient selection, imaging, vascular access, transseptal puncture, valve deployment and assessment of the iatrogenic atrial septal defect at the end of the procedure. Below we provide practical procedural considerations for these steps.

Pre-procedural CT imaging is important to assess the dimensions and characteristics of the previously implanted prosthesis, the surrounding aortomitral anatomy and to simulate the effects of valve implantation during ViV or ViR. CT imaging analysis provides the true internal diameter of the mitral prosthesis and, in cases of ViR, determines whether the ring is complete or incomplete, which may impact upon selection of valve size and implantation strategy. The ViV mitral application provides characteristics and the profile of various surgical prostheses and can be used to guide the selection of appropriate THV prosthesis [73].

#### 4.2.1. LVOT Obstruction

LVOT obstruction is conventionally defined as an increase in LVOT gradient by 10 mmHg, although a clinical impact is more likely evident when the gradient increases by more than 30 mmHg [74,75]. Two principal mechanisms have been described. Fixed LVOT obstruction occurs when the anterior mitral valve leaflet is pushed towards the interventricular septum creating a narrowed and elongated neo-LVOT. In contrast, dynamic LVOT obstruction arises due to systolic anterior motion (SAM) of the anterior mitral valve leaflet towards the interventricular septum during systole, due to forces generated from within the neo-LVOT.

**Figure 5:**
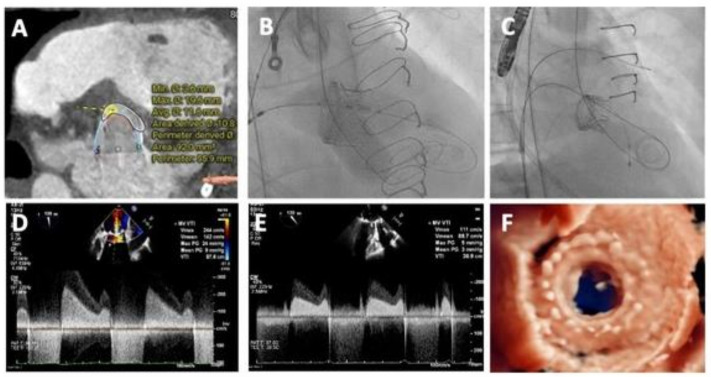
TAVI for treatment of a degenerated 25 mm Perimount surgical bioprosthetic mitral valve. (**A**) 3mensio^TM^ analysis demonstrating the risk of significant LVOTO with a simulated 23 mm Edwards Sapien Ultra S3 at 80/20 deployment (neo-LVOT area 92 mm^2^). (**B**) Kissing balloon inflation with 18 × 45 mm True Dilatation balloon in LVOT prior to full deployment of 23 mm valve. (**C**) final angiographic result. (**D**) Echocardiographic images demonstrating pre-deployment valve gradient, (**E**) post-deployment valve gradient and (**F**) final transoesophageal echo result.

Given that LVOT obstruction is associated with high rates of mortality or the need for open-heart surgery, risk prediction using pre-procedural CT is critical [76] (Figure 5). The risk of developing a fixed LVOT obstruction can be estimated by simulating what impact valve implantation might have on the anterior mitral valve leaflet and subsequent neo-LVOT geometry. A predicted, the cross-sectional area for the neo-LVOT can be calculated, with an area < 200 mm^2^ considered to represent a high risk for the development of a LVOT obstruction [77]. Different valve sizes and implantation depths can then be simulated to see how they may impact upon the predicted neo-LVOT geometry and subsequent risk of obstruction. In contrast, predicting dynamic obstruction is more challenging and depends on multiple anatomical and physiological parameters, which include: aortomitral angulation, mitral annulus-to-interventricular septal distance, basal septal bulge and length of anterior mitral valve leaflet. Additionally, the LVOT obstruction risk is greater for pericardial valves and taller-frame valves than porcine or shorter-frame valves [76,78].

If the risk of LVOT obstruction is deemed to be high, and a conservative or surgical strategy is not feasible, then various adjunctive techniques can be performed. In the first instance, the implantation depth of the valve can be adjusted, with a more ‘atrial’ deployment leading to a potentially larger neo-LVOT (although this does increase the risk of a residual supra-skirt leak). In cases where an enlarged or prominent basal septum may contribute to the LVOT obstruction risk, alcohol septal ablation or radiofrequency ablation may be performed [79,80]. More recently, the SESAME (SEptal Scoring Along the Midline Endocardium) procedure was developed, which mimics surgical myectomy using transcatheter solutions [81].

The alternative target to the basal septum is the anterior mitral valve leaflet, which can be modified or displaced using transcatheter electrosurgical techniques. The LAMPOON (Laceration of the Anterior Mitral leaflet to Prevent Outflow ObstructioN) procedure aims to split the anterior mitral leaflet, deflecting it away from the LVOT and allowing anterograde blood flow through the open cells of the THV frame [77]. If these adjunctive procedures are deemed necessary, these are usually performed up-front, prior to implantation of the THV.

#### 4.2.2. Procedural Steps

Transseptal puncture should ideally be posterior-inferior and be at least 3.5 cm from the mitral valve annulus, performed under transoesophageal echocardiographic guidance. For valve deployment, ensure the Edwards Sapien should be loaded in the correct orientation, 180 degrees opposite to conventional TAVI. A Safari wire (Boston Scientific Corp., Marlborough, MA, USA) is advanced across the mitral valve into the apex of the left ventricle (Figure 5). You may consider positioning a second buddy wire across the atrial septum, this both facilitates the advancement of the system across the septum and obviates the requirement to place a temporary pacing wire that is conventionally required for rapid ventricular pacing [82]. The septum needs to be balloon dilated (according to the delivery system size, usually 14 mm) to enable the THV delivery system to pass. The THV is deployed at a slow rate (not necessarily requiring ventricular pacing). Following valve deployment, transoesophageal echocardiography is used to assess valve position, motion of the leaflets, trans-mitral gradients, presence of paravalvular leaks, and gradient across the LVOT. After removal of the delivery system, assess the iatrogenic septal defect for the presence of a significant right-to-left inter-atrial shunt or pulmonary hypertension with right ventricular failure. If required, the iatrogenic septal defect can be closed with a septal closure device.

### 4.3. Future Perspectives

Globally, the prevalence of significant mitral regurgitation is much greater than severe aortic stenosis. Therefore, the field of TMVR is set to expand, particularly with the emergence of dedicated TMVR devices and solutions for leaflet modification. Further evolutions in imaging technologies with the development of 3D modelling, 3D printing and patient-specific computational modelling is further expected to improve the prediction of LVOT obstruction and guide valve implantation strategies to ensure optimal lifelong results.

## 5. TAVI for Treating Degenerated Tricuspid Bioprosthesis

Approximately 5400 tricuspid valve surgeries are performed annually in the United States. A substantial percentage of these require reoperation due to valve degeneration or regurgitation, especially as tricuspid valve prostheses typically exhibit shorter lifespans compared to systemic valvular prostheses [83,84,85]. The mortality rates associated with tricuspid valve reoperations are notably high, with retrospective data indicating elevated risks, especially concerning operative mortality. The emergence of transcatheter tricuspid valve-in-valve (TVIV) and valve-in-ring (TVIR) implantation presents an encouraging alternative for managing failed tricuspid bioprostheses, particularly crucial due to the escalated surgical risks associated with redo tricuspid surgery. In cases involving right ventricular (RV) dysfunction, often linked to a failed tricuspid prosthesis, the safety profile and success rate of TVIV and TVIR contrast starkly with the complexities of reoperation [86,87].

These advancements align with recent guidelines from the American College of Cardiology (ACC) and the American Heart Association (AHA), endorsing catheter-based treatments for high-risk patients with prosthetic valve dysfunction. Therefore, in this section we discuss the principles, techniques, and considerations for TViR and TViV procedures.

### 5.1. Comprehensive Procedural Guide for TViV and TViR Interventions

Tricuspid valve interventions demand meticulous planning for successful outcomes. Transthoracic echocardiography (TTE) serves as the primary diagnostic tool, particularly for assessing degenerated tricuspid prostheses and failed ring repairs. Recent guidelines define clinically significant tricuspid stenosis with a mean transvalvular gradient of ≥5 mmHg; however, in tricuspid bioprostheses, a mean gradient ≥10 mmHg may better signify significant stenosis. Nonetheless, transvalvular gradients are heart rate dependent and may elevate due to patient–prosthesis mismatch. Hence, comparison with previous examinations is vital for accurate differentiation between prosthesis stenosis, valve thrombosis, or mismatch issues [1,83,88,89].

### 5.2. Preprocedural Assessment

A multi-modality imaging approach is paramount to ensure an accurate assessment of the anatomy and physiology of the tricuspid valve complex as well as to plan and simulate any valve intervention.

#### 5.2.1. Imaging Modalities

Transthoracic echocardiography (TTE) serves as the initial step and offers insights into valve structure, function and haemodynamics [83,88]. CT adds a layer of intricate anatomical detail, offering three-dimensional reconstructions. It aids in evaluating annular dimensions, prosthesis orientation, and relationships with adjacent structures [90]. Cardiac magnetic resonance imaging (CMR) is particularly useful in scenarios where other imaging modalities may present limitations. CMR complements the evaluation of right ventricular function, valve morphology, and pulmonary vasculature [83].

#### 5.2.2. Planning Considerations

Accurate valve sizing and selection according to manufacturer specifications is critical. Different valves like the Melody (Medtronic, Minneapolis USA) and Sapien 3 (Edwards Lifesciences, Irvine, CA, USA) offer varying characteristics suited to different anatomical configurations. The Melody valve, suitable for diameters up to 25 mm, may require different deployment techniques compared to the Sapien 3, which can accommodate larger diameters up to 31 mm due to its longer leaflets and stent height [1,91].

#### 5.2.3. Computed Tomography (CT) for Anatomical Assessment

Three-dimensional reconstruction for procedural planning: CT imaging does not just offer detailed anatomical insights but also allows for three-dimensional reconstructions. Specialized software facilitates the creation of precise anatomical models, mimicking transcatheter heart valve implantation. This advanced simulation aids in meticulous procedural planning, predicting potential challenges and optimizing strategies for valve deployment [87,92].

#### 5.2.4. Valve Deployment

Understanding the capability of valves like the Sapien 3 to undergo controlled overexpansion is vital. This feature allows a broader range of annular diameters to be accommodated, ensuring an optimal fit and reducing the risk of paravalvular leaks [93]. Determining the ideal position of the THV concerning the surgical valve stent frame or the annulus plane is critical. Various markers and landmarks help guide precise deployment, reducing the likelihood of migration or improper placement [87,92].

### 5.3. Procedural Considerations for TViVs and TViRs

#### 5.3.1. Vascular Access and Procedural Approach

Vascular access is often performed via the jugular or femoral vein. The choice between these access points depends on the tricuspid valve orientation. Modern delivery systems offer versatility, allowing femoral access despite oblique valve positioning. Alternatively, a transatrial approach might be considered but is more complex and typically reserved for specific conditions, such as challenging venous anatomy or inaccessible routes [94,95]. Conscious sedation and local anaesthesia are commonly employed but may hinder the use of transoesophageal echocardiography (TOE) during the procedure, impacting intraprocedural reassessment and optimal positioning of wires and the prosthesis [90].

#### 5.3.2. Crossing the Tricuspid Valve

**Figure 6:**
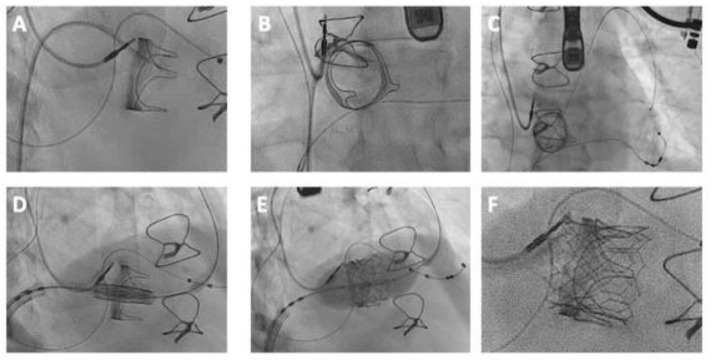
TAVI for treatment of a degenerated 25 mm Carpentier-Edwards bioprosthetic valve implanted in the tricuspid position. (**A**,**B**) Valve crossed with an Amplatz Left catheter and straight-tipped 0.035″ wire, which was (**C**) positioned in the pulmonary artery. (**D**,**E**) Implantation of a 26 mm Edwards Sapien S3 Ultra valve with (**F**) good final result on angiography.

Navigating the tricuspid valve can be challenging, especially with a small valve orifice or severely dilated right atrium. Adjusting fluoroscopy helps provide an optimal view of the prosthesis, facilitating catheter guidance (Figure 6). Employing a multipurpose catheter with a shaped wire aids in directing the catheter toward the tricuspid valve. Additionally, using a steerable sheath in combination with an angled wire ensures a secure catheter position during guidewire deployment into the right ventricular apex [73,94,95].

#### 5.3.3. Wire Positioning

Upon successful traversal of the tricuspid valve, the wire is most commonly positioned in the pulmonary artery for extra support, but right ventricular apical positioning is also possible (Figure 6).

#### 5.3.4. Valve Sizing and Selection

The choice of valve size, whether it is the Melody valve or the Sapien 3, is contingent upon the diameter of the surgical prosthesis or ring. Guidelines specify that a Melody valve is suitable for a surgical prosthesis with a diameter of ≤25 mm, whereas a Sapien 3 valve is recommended for a prosthesis of ≥29 mm.

Precise positioning of the transcatheter heart valve is crucial for optimal function and avoidance of complications. Specific guidelines outline the proper alignment of markers or indicators based on the characteristics of the existing prosthesis or annuloplasty ring to achieve accurate valve placement and minimize the risk of complications like paravalvular leakage or malpositioning [90].

### 5.4. Valve Implantation

Before the procedure, thorough preparations involve ensuring the valve is appropriately mounted onto the catheter. Precise orientation during the introduction of the valve into the sheath is pivotal for successful placement [90,96]. Implantation techniques vary based on the type of valve being used, such as the Melody valve or the Sapien 3, and are influenced by several critical factors:

- Valve Preparation: Mounting the valve onto the delivery catheter involves a series of steps ensuring the correct orientation of the valve. For instance, the Melody valve is positioned as per standard protocols used in pulmonary valve implantation [97].
- Delivery Catheter Orientation: Ensuring the delivery catheter is appropriately oriented before introducing the valve into the sheath is crucial. For the Sapien 3 valve, the Edwards E logo orientation downward facilitates the correct flexion of the catheter during placement [89,98].
- Implantation Techniques: Deploying the valve involves a methodical approach tailored to the specific valve being used. The Melody valve typically requires dilatation of the inner balloon, ensuring approximately 40% of the stent frame is aligned within the right atrium. Subsequent inflation of the outer balloon aligns the valve into the correct tricuspid position. Conversely, the Sapien 3 valve may necessitate overexpansion up to a diameter of 31 mm, thanks to its design features like longer leaflets and a taller stent height. Proper positioning concerning the surgical valve stent frame or other markers is critical for optimal deployment [90,92].
- Post-Implantation Assessment: Evaluation following valve implantation is imperative. Utilizing imaging modalities like transoesophageal echocardiography (TOE) or other imaging systems allows for real-time assessment to confirm correct positioning, function and absence of complications [96,97].
- Balloon Sizing and Positioning: In challenging cases involving very stenotic valves, pre-dilation of the prosthesis might be considered to facilitate subsequent valve deployment. Specific guidelines dictate the positioning of the central marker of the Sapien 3 valve concerning the surgical valve stent frame or other anatomical markers, ensuring accurate alignment and minimizing the risk of malposition or paravalvular leakage [73].
- Procedural Modifications: Adjustments during the procedure might be necessary based on real-time observations. Techniques like retracting the Sapien 3 delivery catheter pusher or slightly inflating the balloon for better tracking of the system can aid in overcoming obstacles encountered during valve crossing or deployment [99].

### 5.5. Future Perspectives

Tricuspid valve-in-valve (TViV) and tricuspid valve-in-ring (TViR) interventions offer safer alternatives to conventional redo tricuspid valve surgeries. Both techniques exhibit high technical success rates and clinical efficiency. While TViV procedures exhibit consistent success rates with minimal complications, TViR poses challenges due to the anatomical complexities associated with tricuspid annuloplasty rings/bands. This complexity often leads to higher rates of valve malposition and paravalvular leaks, making TViR technically more demanding. Future developments in orthotopic valve replacements might pave the way for a shift from off-label use of balloon-expandable valves to clinically developed orthotopic valves for TTVR (transcatheter tricuspid valve replacement).

## 6. Conclusions

In summary, the improvements in procedural techniques and device technologies have expanded the potential applications for TAVI. Patients with degenerated bioprosthetic aortic, mitral or tricuspid valves would conventionally only have two management strategies available: conservative treatment or redo surgery, which can be complicated and is associated with higher mortality and peri-operative complications. Therefore, the possibility of treating these degenerated bioprosthesis with TAVI remains an attractive management strategy. Further refinements in procedural techniques combined with iterative improvements in device technologies or dedicated valve technologies are anticipated as the field of TAVI and its potential applications continues to grow.

## Figures and Tables

**Table 1:** Valve-in-valve data.

Data on ViV Outcomes					
Authors	Year	No. of Patients	Patient Cohort	Procedures Performed	Main Outcomes
Dvir, D., et al. [6]	2014	459	VIVID Registry (prospective, multinational, 55 centers, all-comer)	BEV (Sapien) and SEV (mainly Evolut) ViV	30-day mortality 7.6%, 1.7% stroke; 1-year mortality 16.8%, higher mortality in patients with smaller valves and predominant stenosis of SAVR valve
Webb, J.G., et al. [7]	2017	365	PARTNER 2 ViV Registry (multicenter, continued access)	BEV (using 23- or 26 mm Sapien XT)	30-day mortality 2.7%, 2.7% stroke; 1-year mortality 12.4%, mean gradient 17.6 mmHg, ≥moderate PVL 1.9%
Deeb, G.M., et al. [8]	2017	233	CoreValve U.S. Expanded Use Study (prospective, nonrandomized)	SEV (23- to 31 mm CoreValve)	30-day mortality 2.2%, 0.4% stroke; 1-year mortality 14.6%, mean gradient 16.6 mmHg (higher gradients in smaller SAVR, stenosis as mode of failure, patient prosthesis mismatch)
Tuzcu EM et al. [9]	2018	1150	STS/ACC Registry (consecutive patients undergoing TAVI in US; 1:2 matched with native TAVI)	BEV and SEV ViV	30-day mortality 2.9%, 1.7% stroke; 1-year mortality 11.7%; higher gradients (16 vs. 9 mmHg; highest in small & stenotic SAVRs) compared with native TAVI, but less moderate/severe AR (3.2% vs. 6.6% in native TAVI)
Randomized Data on ViV Outcomes					
Rodés-Cabau, et al. [13]	2022	102	Patients with small SAVR (≤23 mm) randomized between BEV (n = 46) & SEV (n = 52)	BEV and SEV ViV	Similar clinical outcomes (no death, no stroke) at 30 days; lower mean gradients in SEV vs. BEV (15 vs. 23 mmHg; on echo but not via invasive hemodynamics), tendency to less PPM (44% vs. 64%)

ViV = valve-in-valve; STS = Society of Thoracic Surgeons; ACC = American College of Cardiology; TAVI = Transcatheter Aortic Valve Implantation; BEV = balloon-expandable valve; SEV = self-expandable valve; AR = aortic regurgitation; PPM = permanent pacemaker; PVL = paravalvular leak.

**Table 2:** Summary of the literature reporting procedural and clinical outcomes of redo TAVR.

Author	Study Period	Cohort	Redo TAVI as % of Total TAVI	Index THV	Redo THV Type	THV Failure	Reported Outcomes
Barbanti et al., 2016 [36]	2014–2016	Redo-TAVI 14 centers N = 50	50/13,876 (0.4%)	SEV: 92% BEV: 8%	SEV: 60% BEV: 40%	PVL: 50% Pure AR: 26% Pure AS: 18% Combined AS/AR: 6%	In-hospital Mortality: 0% Stroke: 2% PPM: 8.6%
Landes et al., 2020 [41]	2008–2021	Redo-TAVR 37 centers N = 212	212/63,876 (<0.01%)	SEV: 61% BEV: 39%	SEV: 50% BEV: 50%	Pure AR: 44.8% Pure AS: 29.7% Combined AS/AR: 25.4%	Peri-procedural Stroke: 1.4% Malposition: 3.3% CO: 0.9% PPM: 9.6% 30-day mortality: 2.8% 1-year mortality: 13.5%
Testa et al., 2021 [37]	2008–2020	TRANSIT 28 centres N = 172	172/40,000 (<0.01%)	SEV: 65% BEV: 35%	SEV: 61%	Pure AR: 56% Pure AS: 33% Combined AS/AR: 10%	In-hospital Mortality: 4.1% Stroke: 3.5% PPM: 8.6% MI: 1.2% 30-day mortality: 7%
Percy et al, 2021 [39]	2012–2017	US Medicare N = 617	n = 617/133,650 (<0.01%)	N/A	N/A	N/A	30-day mortality: 6.2% 1-year mortality: 21%
Tanget al, 2023 [38]	2009–2022	EXPLANT OR REDO-TAVR 29 centres N = 215	215/66,760 (<0.01%)	SEV: 54% BEV: 40% MEV: 6%	SEV: 47% BEV: 50% MEV: 3%	SVD: 63.7% PVL: 32.8% PPM: 0.5% PVT: 0.39% Delayed migration: 0.5%	In-hospital Mortality: 2.8% PPM: 11.1% Stroke 3% CO: 0.5% 30-day mortality: 8% 1-year mortality: 22.3%
Makkar et al., 2023 [42]	2011–2022	STS/TVT registry All BEVN = 1320	1320/350,591 (<0.01%)	SEV: 61% BEV: 39%	BEV: 100%	Mod-severe AR: 64.8% Mean AV gradient: 36.7	In-hospital Mortality: 3.4% Stroke 1.6% PPM: 6.1%

TAVI = transcatheter aortic valve implantation; BEV = balloon-expandable valve; SEV = self-expandable valve; AS = aortic stenosis; AR = aortic regurgitation; PPM = permanent pacemaker; PVL = paravalvular leak; CO = coronary obstruction; N/A = not available; MEV = mechanically expandable valve; SVD = structural valve deterioration; PVT = prosthetic valve thrombosis; AV = aortic valve.

## Data Availability

The data presented in this study are available on request from the corresponding author.
